# EfficientNet-CICA: a sports image classification model based on causal intervention channel attention

**DOI:** 10.3389/fphys.2026.1905347

**Published:** 2026-10-12

**Authors:** Junnan Yang, Shaocong Zhao

**Affiliations:** The Department of Physical Education, Xiamen University of Technology, Xiamen, China

**Keywords:** channel attention, deep learning, exercise physiology, sports-context recognition, visual phenotyping

## Abstract

Accurate recognition of sport and exercise contexts from static visual data can provide an automated annotation component for multimodal sport and exercise analysis, but it should be distinguished from direct physiological measurement. Such recognition is challenging because sports images often contain large variations in posture, equipment, viewpoint, occlusion, and environmental background, and visually similar activities may share overlapping movement cues. In this study, we developed an EfficientNet-based sports-context recognition framework with a Causal Intervention Channel Attention (CICA) mechanism. The proposed module uses class prototype information and counterfactual channel intervention to recalibrate feature responses toward category-discriminative regions, including athletes, equipment, body-action patterns, and representative exercise environments. Experiments were performed on a public 22-class sports image dataset containing 14,192 valid images. Across five random seeds, EfficientNet-CICA achieved an accuracy of 0.9456 ± 0.0060, a weighted F1-score of 0.9455 ± 0.0059, and a macro-average AUC of 0.9986 ± 0.0003. Paired statistical analysis showed consistent improvements over the EfficientNet baseline and conventional attention variants, whereas the margin over MedMamba was smaller and was therefore interpreted cautiously. Ablation experiments confirmed the contribution of prototype-guided channel intervention, while confusion matrices, ROC and precision–recall curves, t-SNE visualization, and Grad-CAM analysis showed improved feature separability and more interpretable attention to sport-relevant visual evidence. To address the physiological motivation more explicitly, we added two auxiliary analyses: a toy sport-aware rulerouting demonstration and a PAMAP2 heart-rate regression experiment, in which adding activity type to IMU features reduced MAE from 4.39 to 3.91 bpm. These analyses suggest that activity context can influence downstream physiological interpretation, while the proposed image model itself remains a static-image visual classifier. Our code are available in: https://github.com/xingshdlsjz-dev/CICA.

## 1 Introduction

This study focuses on static-image sports-context classification. It does not collect or directly model physiological, biomechanical, or wearable signals in the main image-classification experiment. Exercise physiology increasingly relies on contextual information to interpret physiological responses during physical activity. Training-load research has emphasized that physiological and biomechanical load-adaptation pathways should be considered separately (Vanrenterghem et al., 2017). It has also been argued that internal-load measures need to be interpreted in relation to external activity demands and movement context (Halson, 2014; Bourdon et al., 2017). In addition, physical activity guidelines distinguish aerobic, vigorous-intensity, and muscle-strengthening activities, indicating that different exercise types involve different physiological demands and health-related adaptations (Bull et al., 2020). Accordingly, recognizing sport type may provide an activity-context variable for downstream physiological interpretation, for example by selecting activity-specific thresholds or routing a sample to a sport-aware model. However, such use should be regarded as a hypothesis for multimodal systems unless physiological outcomes are tested directly. With the expansion of sports broadcasting, mobile cameras, wearable devices, and intelligent training platforms, image-based sports classification may therefore support sport-specific data organization and future multimodal systems that combine visual context with physiological, biomechanical, or wearable signals (Camomilla et al., 2018; Aroganam et al., 2019).

Despite this potential, visual recognition of sports contexts remains difficult. Sports images are characterized by complex backgrounds, large variations in body posture, motion-related blur, occlusion, diverse camera viewpoints, and strong visual similarity among categories. Existing sports image and video recognition datasets have shown that sports scenes often contain heterogeneous visual cues and category overlap (Moreira et al., 2016; Podgorelec et al., 2020). General action-recognition benchmarks further highlight the difficulty of recognizing activities under diverse viewpoints and backgrounds (Kuehne et al., 2011; Soomro et al., 2012). For example, tennis and badminton may share racket-like equipment and court layouts, wrestling and WWE may contain similar body-contact patterns, and hockey and ice hockey may share visually similar player–equipment structures, as also reflected in early sports-action recognition settings (Rodriguez et al., 2008). In exercise physiology or sport monitoring scenarios, such errors may lead to inaccurate contextual annotation of training or competition records. Thus, models are required not only to extract global scene information, but also to focus on category-discriminative cues such as athletes, sport-specific equipment, posture patterns, and movement-related local regions.

Deep learning has substantially advanced image classification and visual representation learning. Large-scale image recognition was strongly accelerated by ImageNet and early deep CNNs (Deng et al., 2009; Krizhevsky et al., 2012). Subsequent convolutional architectures, including VGG and ResNet, further improved hierarchical visual feature extraction through deeper network designs (He et al., 2016; Ishfaque et al., 2026). Inception networks introduced multi-scale convolutional representations, while EfficientNet improved the balance among network depth, width, and input resolution (Szegedy et al., 2015; Tan and Le, 2019). Later Inception variants, DenseNet, lightweight MobileNet models, and ConvNeXt also contributed to efficient and high-performing visual classification (Szegedy et al., 2016; Huang et al., 2017). Mobile architectures are particularly relevant for efficient deployment on resource-limited devices (Howard et al., 2017; Sandler et al., 2018), whereas ConvNeXt reflects a modernized convolutional design inspired by recent vision architectures (Liu et al., 2022). More recently, self-attention-based architectures such as ViT, DeiT, and Swin Transformer have improved global representation modeling by capturing long-range dependencies (Dosovitskiy et al., 2020; Touvron et al., 2021). Their development builds on the broader success of attention mechanisms in sequence modeling and hierarchical vision modeling (Vaswani et al., 2017; Liu et al., 2021). State space models have also been explored as efficient alternatives for visual sequence modeling (Gu and Dao, 2023; Liu et al., 2024), including medical visual representation learning (Yue and Li, 2024). Attention mechanisms such as SE and CBAM have been widely used to enhance important channels or spatial regions (Hu et al., 2018; Woo et al., 2018). ECA and Coordinate Attention further improve channel interaction and positional encoding in lightweight attention designs (Wang et al., 2020; Hou et al., 2021). Non-local and BAM modules provide additional examples of attention-based context modeling (Park et al., 2018; Wang et al., 2018). However, these methods are usually optimized for generic visual discrimination and may not explicitly separate activity-relevant cues from background or dataset-related confounders. A key requirement for sports-context recognition in exercise physiology applications is interpretability. Visual models should identify sport-relevant evidence, such as the athlete, implement, field or court structure, and characteristic movement configuration, rather than relying on spectators, clothing colors, logos, or background textures. Existing channel attention modules mostly recalibrate features using statistics from a single image and therefore lack explicit category-level semantic guidance. Prototype learning provides a useful mechanism for introducing class-level contrast into representation learning (Snell et al., 2017). Causal representation learning further offers a theoretical basis for reducing sensitivity to spurious correlations and emphasizing more stable explanatory factors (Neuberg, 2003; Schölkopf et al., 2021). Fine-grained visual recognition studies also suggest that discriminative local interactions can help distinguish visually similar categories (Lin et al., 2015; Fu et al., 2017). In this setting, comparing a sample with visually similar but semantically different categories may help the model identify the channels that are most relevant for distinguishing one sport context from another.

To address these issues, we propose an EfficientNet-based framework with a Causal Intervention Channel Attention mechanism. The proposed CICA module introduces class prototypes and constructs a counterfactual descriptor using the most similar negative prototype. This design encourages the network to enhance channels that distinguish the current sports context from visually confusing alternatives, while suppressing irrelevant background responses. We evaluate the method on a public 22-class sports image dataset and compare it with representative CNN, Transformer, state space, and attention-based baselines. In addition to accuracy, F1-score, and AUC, we use confusion matrices, ROC and precision–recall curves, t-SNE, and Grad-CAM to examine model separability and interpretability.

The main contribution of this study is a category-aware visual recognition framework that can improve the automated annotation of sports and exercise contexts.

- We propose a prototype-guided Causal Intervention Channel Attention (CICA) module for finegrained sports-context recognition.
- From the perspective of internal module design, CICA introduces a class prototype memory bank, selects the most similar negative prototype as a confusing semantic reference, and constructs a counterfactual channel descriptor to guide channel recalibration. This design encourages the model to emphasize category-discriminative sports cues while suppressing less relevant background responses.
- We conduct comprehensive comparison experiments, ablation studies, repeated-seed analyses, efficiency measurements, statistical tests, visualization analyses, and auxiliary physiology-motivation experiments. The results show that EfficientNet-CICA provides a statistically supported improvement over the EfficientNet baseline and conventional attention variants, while achieving the highest mean accuracy and weighted F1-score among the evaluated models.

## 2 Materials and methods

### 2.1 Dataset characteristics

The experiments are conducted on a public sports image classification dataset containing 22 sports categories. The dataset covers a wide range of sports scenes, including ball games, combat sports, racing, gymnastics, shooting, swimming, and other representative sports activities. As shown in **Table 1**, the dataset contains 14,192 valid images in total after removing 132 ignored or invalid files. The 22 categories include badminton, baseball, basketball, boxing, chess, cricket, fencing, football, formula1, gymnastics, hockey, ice hockey, kabaddi, motogp, shooting, swimming, table tennis, tennis, volleyball, weight lifting, wrestling, and WWE. The number of images in each category ranges from 442 to 919, indicating that the dataset has moderate class imbalance. Among them, badminton contains the largest number of images, with 919 valid samples, while kabaddi contains the fewest images, with 442 valid samples. This dataset is challenging for sports image classification because it contains large intra-class variations and high inter-class similarities. For example, images from the same sports category may differ significantly in athlete pose, camera viewpoint, illumination, background, and competition environment. Meanwhile, some categories share similar visual cues, such as tennis and table tennis, hockey and ice hockey, or wrestling and WWE. These characteristics require the classification model to learn discriminative visual representations from both global scene information and local sports-specific regions, such as athletes, equipment, and motion-related patterns.

**Table 1:** Statistics of the sports image classification dataset.

Class	Images	Ignored	Class	Images	Ignored
Badminton	919	8	Ice hockey	699	6
Baseball	724	0	Kabaddi	442	2
Basketball	483	3	MotoGP	659	10
Boxing	695	0	Shooting	526	1
Chess	470	1	Swimming	677	4
Cricket	658	38	Table tennis	697	6
Fencing	625	1	Tennis	704	3
Football	776	15	Volleyball	691	9
Formula 1	671	6	Weight lifting	562	1
Gymnastics	704	7	Wrestling	591	9
Hockey	560	2	WWE	659	0
Total	14192	132			

The Images column reports the valid samples retained for experiments, and the Ignored column reports invalid or unusable files excluded during data cleaning. The final dataset contains 14,192 valid images from 22 sports categories, with 132 ignored files.

### 2.2 Overall framework

Given an input sports image **I**, we employ EfficientNet-B0 as the backbone network for hierarchical feature extraction. Different from directly using the original EfficientNet backbone, the proposed Causal Intervention Channel Attention (CICA) module is incorporated into the EfficientNet backbone to enhance category-discriminative channel responses during feature learning. By introducing category-aware channel recalibration into the backbone representation learning process, the model can emphasize sports-relevant visual cues while suppressing less informative background responses, thereby improving the discriminative representation ability of EfficientNet for sports image classification. The overall architecture of EfficientNet-CICA is illustrated in **Figure 1**.

**Figure 1:**
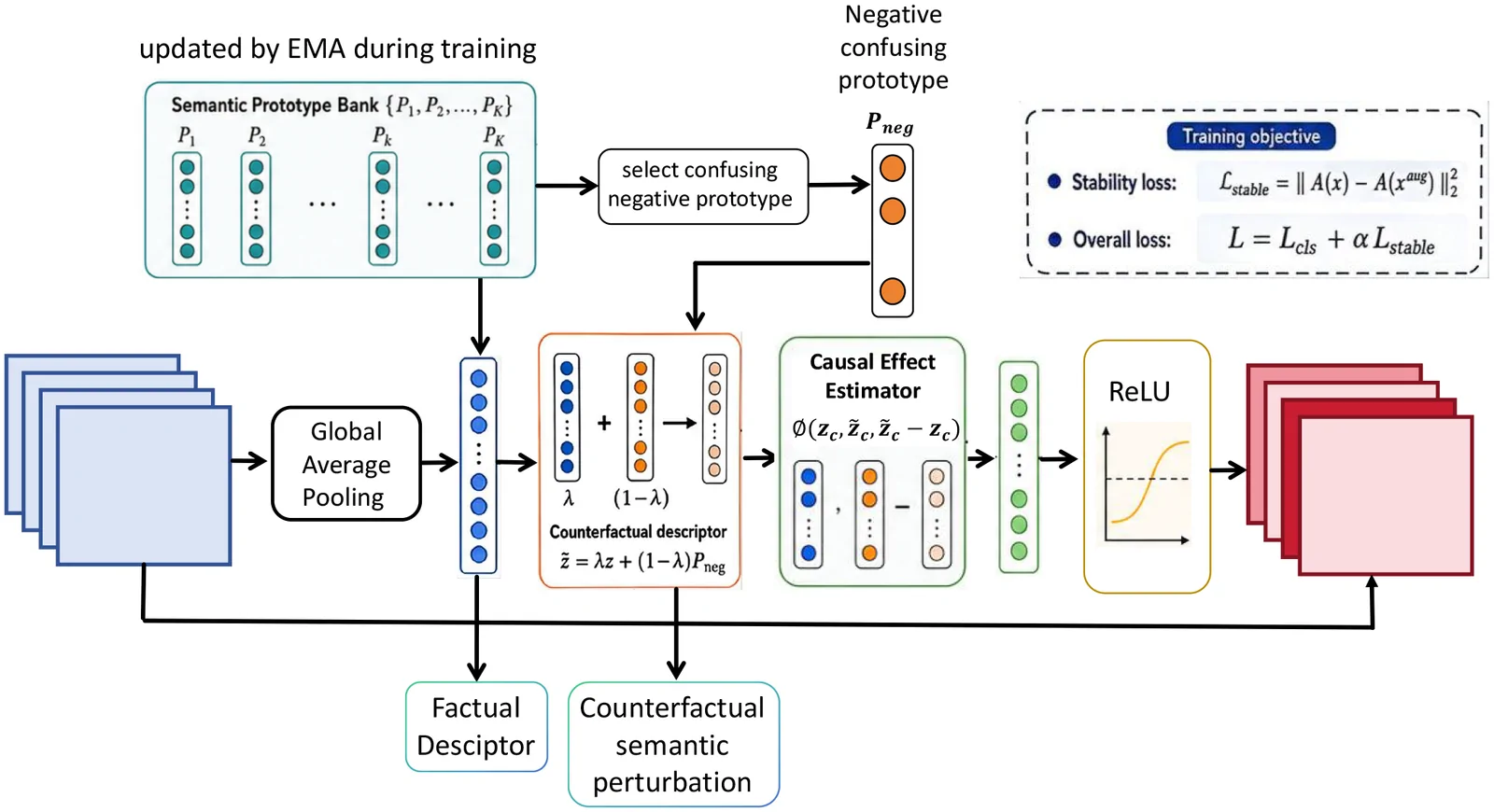
Overall architecture of the proposed EfficientNet-CICA framework. The Causal Intervention Channel Attention (CICA) module is incorporated into the EfficientNet-B0 backbone for category-aware channel recalibration. It converts the backbone feature map into a category-aware channel descriptor, compares it with a confusing negative prototype through counterfactual intervention, generates channel attention weights, and recalibrates feature responses for sports image classification.

Let the feature map generated by the EfficientNet backbone before channel recalibration be denoted as shown in Equation 1:

(1) $$X=f_{\theta }^{pre}\left(I\right)\in {\mathbb{R}}^{C\times H\times W},$$

where *C*, *H*, and *W* represent the number of channels, height, and width of the feature map, respectively. The embedded CICA module performs category-aware channel recalibration on **X** and produces the refined feature representation as shown in Equation 2:

(2) $$\hat{X}=\text{CICA}\left(X\right).$$

The refined feature map $\hat{X}$ is then further transformed within the EfficientNet backbone and subsequently passed through global pooling and a linear classifier for sports category prediction. In this way, the proposed attention mechanism is not treated as an external post-processing component, but is embedded within the backbone feature learning process to enhance category-discriminative sports representations.

### 2.3 Category-aware channel descriptor

Conventional channel attention modules usually rely only on global statistics of the current sample, which may be insufficient for fine-grained sports image classification. In complex sports scenes, different categories may share similar backgrounds, human poses, or visual textures. Therefore, it is important to enhance the channels that are truly discriminative for the target sports category within the backbone feature learning process.

For the feature map **X** extracted from the EfficientNet-B0 backbone, we first apply global average pooling to obtain a channel descriptor as defined in Equation 3:

(3) $$z=\text{GAP}\left(X\right),\;z_{c}=\frac{1}{HW}\sum_{h=1}^{H}\sum_{w=1}^{W}X_{c,h,w},$$

where $z\in {\mathbb{R}}^{C}$ summarizes the global response of each channel.

To introduce category-level guidance into backbone feature recalibration, we maintain a prototype memory bank as defined in Equation 4:

(4) $$P=\left\{p_{1},p_{2},\ldots ,p_{K}\right\},$$

where *K* denotes the number of sports categories and $p_{k}\in {\mathbb{R}}^{C}$ represents the prototype feature of the *k*-th class. The memory bank is initialized from mini-batch descriptors at the beginning of training and then updated online. During training, the prototype of the ground-truth class is updated using an exponential moving average as shown in Equation 5:

(5) $$p_{y}\leftarrow mp_{y}+\left(1-m\right)z,$$

where *y* is the ground-truth label and *m* is the momentum coefficient.

### 2.4 Counterfactual channel intervention

To make the embedded attention module focus on category-discriminative channels, we construct a counterfactual feature descriptor by introducing a confusing negative class prototype. Specifically, for a sample with feature descriptor **z** and label *y*, we select the most similar prototype from other categories according to Equation 6:

(6) $$k^{-}=\arg\underset{k\neq y}{\max}\text{ cos}\left(z,p_{k}\right),$$

where cos(·,·) denotes cosine similarity. The corresponding negative prototype is denoted as $p^{-}=p_{k^{-}}$.

Then, a counterfactual descriptor is generated by mixing the original descriptor and the negative prototype, as defined in Equation 7:

(7) $$z^{cf}=\lambda z+\left(1-\lambda \right)p^{-},$$

where *λ* controls the strength of the intervention. We use the term “counterfactual intervention” in an operational representation-learning sense: the descriptor of the current sample is evaluated under a deliberately substituted semantic reference from a non-ground-truth class, while the remaining backbone computation is kept unchanged. This does not establish causal effects in the physiological sense; rather, it provides a structured hard-negative channel perturbation that approximates the question of which channels would remain discriminative if the sample were contrasted with a visually confusing alternative. This operation encourages the backbone to compare the current sample with visually similar but semantically different categories, thereby identifying channels that are critical for distinguishing fine-grained sports classes.

### 2.5 Channel attention generation

For each channel, we combine the original descriptor, the counterfactual descriptor, and their difference as shown in Equation 8:

(8) $$u_{c}=\left[z_{c},\;z_{c}^{cf},\;z_{c}-z_{c}^{cf}\right].$$

The channel attention weight is generated by a lightweight multilayer perceptron according to Equation 9:

(9) $$a_{c}=\sigma \left(W_{2}\delta \left(W_{1}u_{c}\right)\right),$$

where *δ*(·) is the ReLU activation function, *σ*(·) is the sigmoid function, and *a_c_* ∈ [0,1] denotes the importance score of the *c*-th channel.

The backbone feature map is then recalibrated as shown in Equation 10:

(10) $${\hat{X}}_{c,h,w}=a_{c}\cdot X_{c,h,w}.$$

After channel recalibration, the refined feature map $\hat{X}$ continues through the backbone representation learning process and the classification head, as expressed in Equation 11:

(11) $$o=W_{cls}\cdot \text{GAP}\left(f_{\theta }^{post}\left(\hat{X}\right)\right),$$

where **o** denotes the predicted logits and $f_{\theta }^{post}\left(\cdot \right)$ represents the subsequent feature transformation after CICA-based recalibration. The whole network is optimized using the cross-entropy loss defined in Equation 12:

(12) $${ℒ}_{ce}=-\frac{1}{N}\sum_{i=1}^{N}\text{log }\frac{\text{exp}(o_{i,y_{i}})}{\sum_{k=1}^{K}\text{exp}(o_{i,k})}.$$

The overall training procedure of EfficientNet-CICA is summarized in **Table 2**.

**Table 2:** Training procedure of EfficientNet-CICA.

Step	Operation
1	Initialize EfficientNet-B0 parameters, classifier parameters, and the class prototype memory bank $P\in {\mathbb{R}}^{K\times C}$.
2	For each mini-batch, extract the intermediate feature map ***X*** and compute the channel descriptor z=GAP(X).
3	Update the prototype of the ground-truth class using EMA: $p_{y}\leftarrow mp_{y}+\left(1-m\right)z$.
4	Select the most similar negative prototype: $k^{-}=\arg\;{\text{max}}_{k\neq y}\text{cos}\left(z,p_{k}\right)$.
5	Construct the counterfactual descriptor $z^{cf}=\lambda z+\left(1-\lambda \right)p_{k^{-}}$.
6	Generate channel-wise attention from $\left[z_{c},z_{c}^{cf},z_{c}-z_{c}^{cf}\right]$ using a lightweight MLP and sigmoid activation.
7	Recalibrate the feature map ${\hat{X}}_{c,h,w}=a_{c}X_{c,h,w}$ and continue the remaining EfficientNet-B0 layers.
8	Optimize the classification loss and attention regularization loss by back-propagation.

The pseudocode clarifies prototype initialization, exponential moving-average updating, most-similar negative prototype selection, attention-weight calculation, and integration into EfficientNet-B0.

Bold mathematical symbols denote vector- or tensor-valued quantities used in the model formulation.

### 2.6 Implementation details

All experiments were implemented using PyTorch and trained on an NVIDIA RTX 4090 GPU. The proposed model adopts EfficientNet-B0 as the backbone network, and the proposed CICA module is embedded into the high-level feature extraction stage of the backbone rather than being simply appended after the backbone. For a fair comparison, all baseline models and ablation variants were trained under the same dataset split and optimization settings.

The dataset was divided into training, validation, and testing sets at a ratio of 7:1:2 using stratified sampling. For the original single-run comparison, the random seed was fixed at 42. In response to the reviewer concern regarding run-to-run variability, we additionally repeated the central comparisons using five random seeds: 42, 123, 2024, 3407, and 777. The exact train, validation, and test split files were exported for each run to improve reproducibility. The input image size was set to 224 × 224. During training, random resized cropping, horizontal flipping, and RandAugment were applied for data augmentation (Cubuk et al., 2020). During validation and testing, images were first resized and then center-cropped to 224 × 224.

The models were optimized using the AdamW optimizer, with an initial learning rate of 3 × 10^−4^ and a weight decay of 1 × 10^−4^ (Loshchilov and Hutter, 2017). The batch size was set to 32, and each model was trained for 30 epochs. A cosine annealing learning rate scheduler was adopted to dynamically adjust the learning rate during training. Cross-entropy loss was used as the classification objective. To accelerate training and reduce GPU memory consumption, mixed-precision training was employed. The final model was selected according to the weighted F1-score on the validation set and then evaluated on the test set.

The software environment used PyTorch 2.5.1, torchvision 0.20.1, timm 1.0.27, NumPy 2.0.1, pandas 2.3.3, scikit-learn 1.7.2, SciPy 1.15.3, Matplotlib 3.10.9, CUDA 11.8, and cuDNN 9.1.0 on a single NVIDIA GeForce RTX 4090 GPU. Because mixed-precision GPU training is not bitwise deterministic even when random seeds are fixed, we report repeated-seed means and standard deviations for central claims rather than relying only on single-run point estimates.

For the proposed CICA module, the intervention coefficient *λ* was set to 0.7, which controls the balance between the original feature descriptor and the selected prototype descriptor. The prototype updating momentum was set to 0.9. The attention generation network was implemented as a lightweight multilayer perceptron with a hidden dimension of 16. In addition, the dropout rate before the final classifier was set to 0.2. To improve the stability of attention learning, an attention consistency regularization term was introduced with a weight of 0.05. The main hyperparameter settings are summarized in **Table 3**.

**Table 3:** Implementation details and hyperparameter settings used for model training and evaluation.

Category	Configuration	Setting
Model	Backbone Input resolution	EfficientNet-B0 224 × 224
	Dropout rate	0.2
Training	Data split Optimizer Initial learning rate Weight decay	70%*/*10%*/*20% AdamW 3 × 10^−4^ 1 × 10^−4^
	Batch size	32
	Training epochs	30
	Scheduler	Cosine annealing
CICA	Intervention coefficient *λ* Prototype momentum *m*	0.7 0.9
	Attention hidden dimension	16
	Attention consistency weight	0.05
Evaluation	Loss function Random seed GPU	Cross-entropy loss 42 for the original run; 42,123,2024,3407,777 for repeated-seed analysis NVIDIA RTX 4090
Software	PyTorch/torchvision/timm CUDA/cuDNN scikit-learn/SciPy	2.5.1/0.20.1/1.0.27 11.8/9.1.0 1.7.2/1.15.3

Unless otherwise specified, the same data split, optimizer, learning-rate schedule, batch size, training epochs, and evaluation settings were applied to all comparison models and ablation variants.

## 3 Results

### 3.1 Comparison Models and Evaluation Metrics

To comprehensively evaluate the effectiveness of the proposed EfficientNet-CICA model, we compare it with several representative image classification methods, covering classical convolutional neural networks, Transformer-based architectures, modern convolutional models, and state space model-based methods. All models are trained and evaluated on the same sports image dataset under the same data split and experimental settings, so as to ensure a fair and consistent comparison. Specifically, the comparison models include VGG, ResNet50, InceptionV3, EfficientNet, ViT, Swin Transformer, ConvNeXt, and MedMamba. These baselines are selected to provide a broad comparison across different visual representation learning paradigms. Among them, VGG, ResNet50, InceptionV3, and EfficientNet represent widely used CNN-based classification frameworks, while ViT and Swin Transformer are included as representative Transformer-based models. ConvNeXt is adopted as a strong modern convolutional baseline, and MedMamba is further included as a state-of-the-art visual state space model for comparison. By involving these diverse baseline models, the evaluation can more comprehensively assess the classification performance of the proposed method from multiple perspectives. In particular, the comparison with MedMamba allows us to examine whether EfficientNet-CICA can achieve competitive or superior performance against recent SOTA architectures. Therefore, the experimental results are expected to demonstrate not only the effectiveness of the EfficientNet backbone, but also the additional contribution of the proposed CICA module in improving category-aware feature representation for sports image classification.

To quantitatively assess the classification performance of different models, we adopt accuracy, precision, recall, F1-score, and area under the receiver operating characteristic curve (AUC) as evaluation metrics (El-Ansary et al., 2026; Imani et al., 2026). These metrics evaluate the model from different perspectives and provide a comprehensive measurement of classification ability. Accuracy measures the proportion of correctly classified samples among all test samples, as defined in Equation 13:

(13) $$\text{Accuracy}=\frac{TP+TN}{TP+TN+FP+FN},$$

where *TP*, *TN*, *FP*, and *FN* denote true positives, true negatives, false positives, and false negatives, respectively.

Precision reflects the proportion of correctly predicted positive samples among all samples predicted as defined in Equation 14:

(14) $$\text{Precision}=\frac{TP}{TP+FP}.$$

As defined in Equation 15, recall measures the proportion of correctly predicted positive samples among all actual positive samples:

(15) $$\text{Recall}=\frac{TP}{TP+FN}.$$

The F1-score is the harmonic mean of precision and recall, which provides a balanced evaluation when precision and recall are both important, as defined in Equation 16:

(16) $$\text{F}1-\text{score}=\frac{2\times \text{Precision}\times \text{Recall}}{\text{Precision}+\text{Recall}}.$$

AUC measures the area under the ROC curve and reflects the model’s ability to distinguish between different categories under varying classification thresholds. For the multi-class sports classification task, we compute the one-vs-rest AUC for each category and report the macro-average AUC as defined in Equation 17:

(17) $${\text{AUC}}_{macro}=\frac{1}{K}\sum_{k=1}^{K}{\text{AUC}}_{k},$$

where *K* is the number of sports categories and AUC*_k_* denotes the AUC value of the *k*-th class.

Since the dataset contains multiple sports categories and the number of samples in different categories may be imbalanced, weighted precision, weighted recall, and weighted F1-score are also reported. The weighted metric is calculated as shown in Equation 18:

(18) $$M_{weighted}=\sum_{k=1}^{K}\frac{n_{k}}{N}M_{k},$$

where *M_k_* denotes the metric value of the *k*-th class, *n_k_* is the number of test samples in that class, and *N* is the total number of test samples. These metrics allow a fairer evaluation of model performance across all sports categories.

### 3.2 Comparison experiments and ablation study

**Table 4** presents the performance comparison between the proposed EfficientNet-CICA model and several representative classification models. The compared methods include classical convolutional neural networks, such as VGG, ResNet50, InceptionV3, and EfficientNet, Transformer-based models, such as ViT and Swin Transformer, the modern convolutional architecture ConvNeXt, and the state space model MedMamba. The overlapping EfficientNet-B0 and EfficientNet-CICA entries in **Tables 4**–**6** are now harmonized to the same representative single-run results. The central quantitative conclusions are instead based on the repeated-seed results in **Table 7**. As shown in **Table 4**, the representative EfficientNet-CICA run achieves an accuracy of 0.9405, weighted precision of 0.9410, weighted recall of 0.9405, and weighted F1-score of 0.9404. Compared with the matched EfficientNet baseline, accuracy increases from 0.9154 to 0.9405, a gain of 2.51 percentage points, while weighted F1-score increases from 0.9154 to 0.9404. Compared with other advanced models in the original single-run comparison, EfficientNet-CICA achieved higher accuracy and weighted F1-score than Swin Transformer, ResNet50, and the original EfficientNet baseline. MedMamba obtained a slightly higher macro AUC value in this single run, whereas EfficientNetCICA achieved higher accuracy, weighted precision, weighted recall, and weighted F1-score. Therefore, the comparison with MedMamba should be interpreted as competitive rather than uniformly superior. The results also show that Transformer-based and state space models achieve strong performance. By introducing category-aware channel interaction into EfficientNet, the proposed CICA module enables the network to focus more effectively on discriminative sports-related regions, such as athletes, equipment, and representative motion patterns.

**Table 4:** Performance comparison with representative classification models on the same 22-class sports image dataset using a fixed random seed of 42.

Model	Attention	Accuracy	Precision	Recall	F1-score	AUC
EfficientNet-B0	CICA	**0.9405**	**0.9410**	**0.9405**	**0.9404**	0.9982
MedMamba	–	0.9383	0.9400	0.9383	0.9385	**0.9984**
Swin Transformer	–	0.9348	0.9353	0.9348	0.9348	0.9976
ResNet50	–	0.9260	0.9273	0.9260	0.9263	0.9976
EfficientNet-B0	–	0.9154	0.9165	0.9154	0.9154	0.9967
VGG	–	0.8820	0.8825	0.8820	0.8818	0.9935
InceptionV3	–	0.8742	0.8750	0.8742	0.8741	0.9938
ViT	–	0.8481	0.8506	0.8481	0.8484	0.9916
ConvNeXt	–	0.8330	0.8370	0.8330	0.8333	0.9912

Precision, recall, and F1-score are reported as weighted averages across classes, and AUC is reported as the macro-average one-vs-rest AUC. The best result in each metric column is highlighted in bold, and the second-best result is underlined. The overlapping EfficientNet-B0 and EfficientNet-CICA entries are harmonized with **Tables 5**, **6**; repeated-seed inference is reported separately in **Table 7**.

**Table 5:** Ablation study of different channel attention modules using EfficientNet-B0 as the backbone.

Backbone	Attention module	Accuracy	Precision	Recall	F1-score	Macro AUC
EfficientNet-B0	CICA	**0.9405**	**0.9410**	**0.9405**	**0.9404**	**0.9982**
EfficientNet-B0	CBAM	0.9225	0.9231	0.9225	0.9224	0.9971
EfficientNet-B0	CICA w/o prototype	0.9211	0.9218	0.9211	0.9211	0.9969
EfficientNet-B0	SE	0.9168	0.9179	0.9168	0.9166	0.9965
EfficientNet-B0	None	0.9154	0.9165	0.9154	0.9154	0.9967
EfficientNet-B0	ECA	0.9147	0.9158	0.9147	0.9147	0.9959
EfficientNet-B0	CA	0.9109	0.9124	0.9109	0.9108	0.9942

Precision, recall, and F1-score are weighted averages, while Macro AUC denotes the macro-average one-vsrest AUC. The comparison evaluates whether the proposed CICA module provides additional benefit over standard channel or spatial attention mechanisms. The best result in each metric column is highlighted in bold, and the second-best result is underlined. The shared no-attention and full-CICA entries are harmonized with **Tables 4**, **6**.

**Table 6:** Effect of different prototype selection strategies in the proposed CICA module.

Prototype strategy	Accuracy	Weighted F1	Macro AUC
No prototype	0.9211	0.9211	0.9969
Random negative prototype	0.9211	0.9210	0.9970
Ground-truth positive prototype	0.9376	0.9375	0.9973
Most similar negative prototype	**0.9405**	**0.9404**	**0.9982**

All variants use the same EfficientNet-B0 backbone and training protocol, while only the prototype selection strategy is changed. Accuracy, weighted F1-score, and macro-average AUC are reported to evaluate whether hard negative semantic intervention improves sports-context discrimination. The best results are highlighted in bold. The full-CICA and no-prototype values are harmonized with the corresponding overlapping entries in **Tables 4**, **5**.

**Table 7:** Five-seed robustness analysis of central comparisons.

Model	Accuracy	Weighted F1	Macro AUC
EfficientNet-CICA	0.9456 ± 0.0060	0.9455 ± 0.0059	0.9986 ± 0.0003
MedMamba	0.9413 ± 0.0034	0.9413 ± 0.0034	0.9982 ± 0.0002
EfficientNet	0.9237 ± 0.0024	0.9237 ± 0.0023	0.9970 ± 0.0006
EfficientNet-CICA w/o prototype	0.9220 ± 0.0048	0.9220 ± 0.0047	0.9966 ± 0.0004
EfficientNet-CBAM	0.9195 ± 0.0049	0.9195 ± 0.0048	0.9965 ± 0.0006

Values are reported as mean ± standard deviation across seeds 42, 123, 2024, 3407, and 777.

**Table 5** reports the ablation results of different channel attention modules based on the EfficientNet backbone. To verify the effectiveness of the proposed CICA module, we compare it with several commonly used attention mechanisms, including SE, ECA, Coordinate Attention (CA), and CBAM. In addition, we also evaluate a variant of the proposed method, denoted as CICA without prototype, to further analyze the contribution of the category prototype guidance. As shown in **Table 5**, the proposed EfficientNetCICA achieves the best performance among all attention variants, with an accuracy of 0.9405, weighted precision of 0.9410, weighted recall of 0.9405, weighted F1-score of 0.9404, and macro AUC of 0.9982. Compared with the EfficientNet baseline without attention, CICA improves the accuracy from 0.9154 to 0.9405, yielding a gain of 2.51 percentage points. The weighted F1-score is also increased from 0.9154 to 0.9404, demonstrating that the proposed module can effectively enhance the feature representation ability of the backbone network. Compared with existing attention modules in the single-run ablation, CICA shows a larger performance gain than SE, ECA, CA, and CBAM. CBAM achieves the second-best performance with an accuracy of 0.9225 and weighted F1-score of 0.9224, but it is still lower than CICA by 1.80 percentage points in accuracy. Because small differences among SE, ECA, CA, and the no-attention baseline may fall within run-to-run variability, we avoid over-interpreting their ordering and instead focus on the larger and repeated-seed-supported gain of CICA. The CICA without prototype variant achieves an accuracy of 0.9211, which is higher than SE, ECA, CA, and the baseline model, but significantly lower than the full CICA module. This result confirms the importance of category prototype guidance. By introducing class-level prototype information and constructing counterfactual channel descriptors, the full CICA module can better distinguish visually similar sports categories and suppress irrelevant background responses. Therefore, the ablation study verifies that both the channel intervention mechanism and the category prototype design are essential for improving classification performance.

### 3.3 Repeated-seed robustness, statistical testing, and efficiency analysis

To address the concern that the original results were reported as single-run point estimates, we repeated the central comparisons using five random seeds. The repeated-seed analysis included EfficientNet, EfficientNet-CICA, EfficientNet-CBAM, EfficientNet-CICA without prototype guidance, and MedMamba. As shown in **Table 7**; **Figures 2**, **3**. EfficientNet-CICA achieved the highest mean accuracy and weighted F1-score across the five runs, with an accuracy of 0.9456 ± 0.0060 and a weighted F1-score of 0.9455 ± 0.0059. The EfficientNet baseline achieved 0.9237 ± 0.0024 accuracy and 0.9237 ± 0.0023 weighted F1-score, indicating an average improvement of approximately 2.19 percentage points after adding CICA.

**Figure 2:**
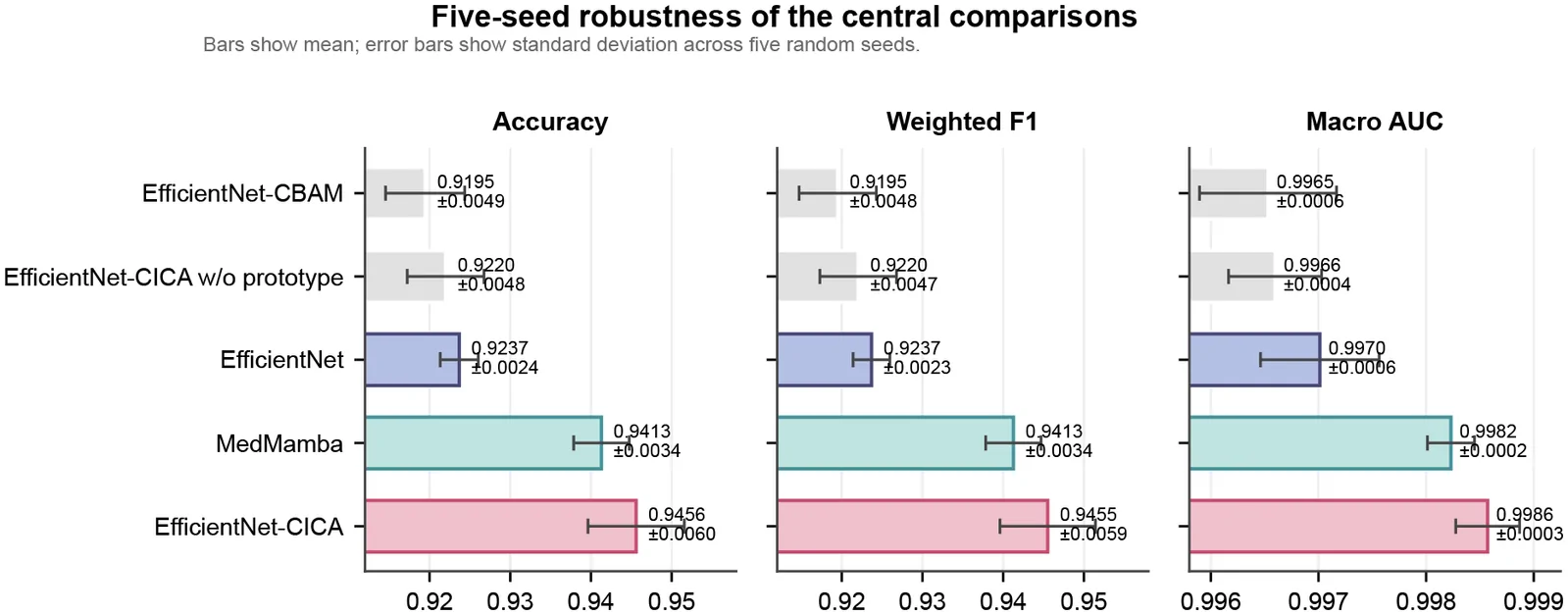
Repeated-seed performance comparison. Bars and error bars show mean and standard deviation across five random seeds.

**Figure 3:**
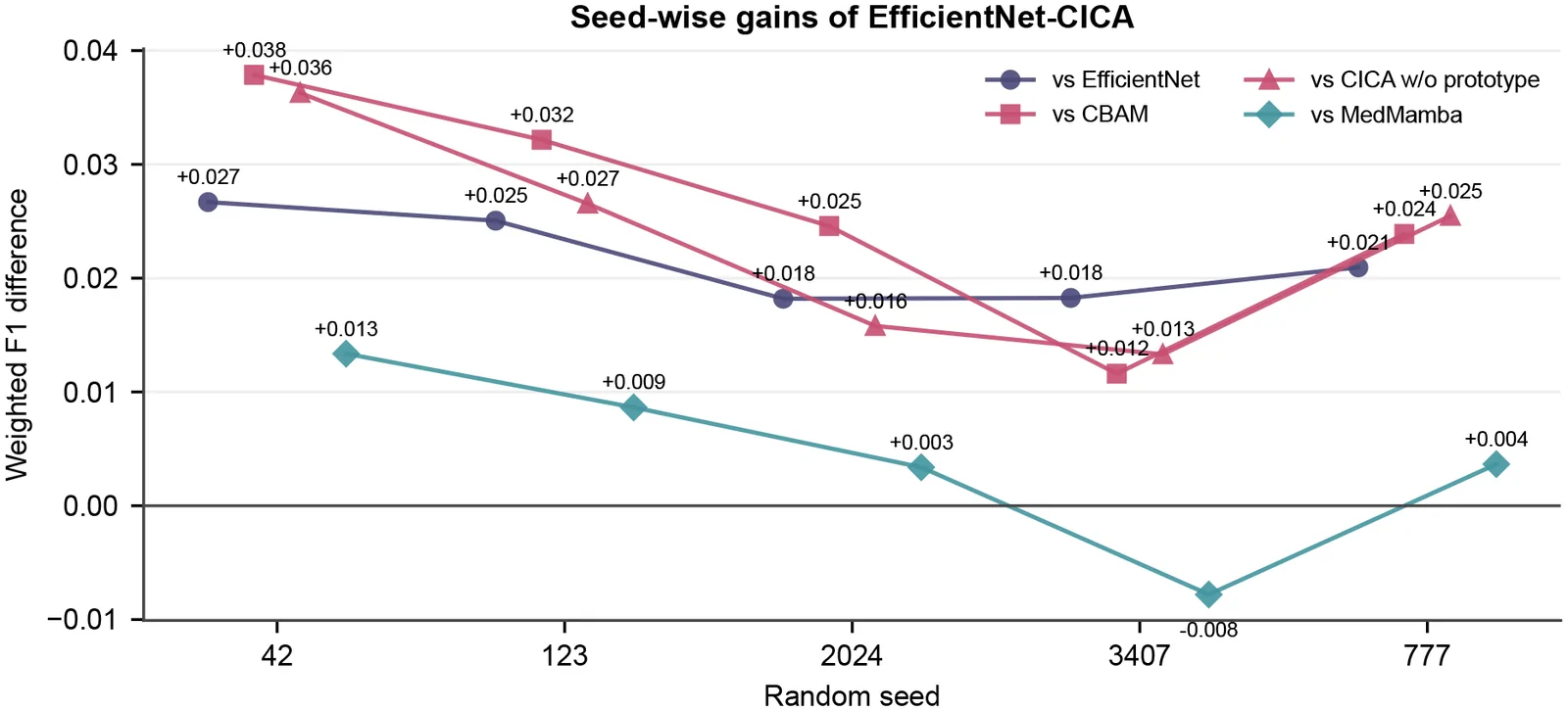
Seed-wise weighted F1-score difference between EfficientNet-CICA and selected baselines. Positive values indicate higher weighted F1-score for EfficientNet-CICA.

We further performed paired statistical comparisons using test-set predictions from matched seeds. **Table 8** summarizes the median McNemar *p* value and the mean accuracy/F1 differences. EfficientNet-CICA showed statistically supported improvements over EfficientNet, EfficientNet-CBAM, and EfficientNetCICA without prototype guidance. In contrast, the difference between EfficientNet-CICA and MedMamba was small, with a median McNemar *p* value of 0.09. Therefore, we revised the interpretation and no longer claim uniform superiority over MedMamba; instead, we state that EfficientNet-CICA achieved the highest mean accuracy and weighted F1-score while remaining competitive with MedMamba.

**Table 8:** Summary of paired statistical comparisons across five seeds.

Comparison	Median McNemar *p*	ΔAccuracy	ΔF1
CICA vs. CBAM	1.977 × 10^−8^	0.0261	0.0261
CICA vs. CICA w/o prototype	2.321 × 10^−9^	0.0237	0.0235
CICA vs. EfficientNet	8.287 × 10^−7^	0.0219	0.0220
CICA vs. MedMamba	0.09	0.0043	0.0042

Positive differences indicate higher performance for EfficientNet-CICA.

Because EfficientNet was selected partly for its efficiency profile, we also measured parameter count, estimated FLOPs, inference latency, throughput, and checkpoint size for EfficientNet attention variants. As shown in **Table 9** and **Figure 4**, CICA increased the parameter count only from 4.0357M to 4.0358M and increased estimated FLOPs from 0.7693G to 0.7694G. The single-image latency increased from 4.1875 ms to 4.4253 ms, indicating a modest runtime cost. Compared with CBAM, CICA used fewer parameters and a similar latency range, while providing stronger classification performance in the repeated-seed analysis.

**Table 9:** Efficiency comparison of EfficientNet attention variants.

Model	Params (M)	FLOPs (G)	Latency (ms/img)	Throughput (img/s)	Size (MB)
EfficientNet	4.0357	0.7693	4.1875	5616.8	15.8234
EfficientNet-SE	4.2419	0.7697	4.1791	5627.2	16.6170
EfficientNet-ECA	4.0357	0.7693	4.1185	5653.7	15.8290
EfficientNet-CA	4.1920	0.7721	4.4108	5378.2	16.4290
EfficientNet-CBAM	4.2420	0.7701	4.4615	5480.5	16.6174
EfficientNet-CICA w/o prototype	4.0358	0.7694	4.2787	5573.5	15.9383
EfficientNet-CICA random negative	4.0358	0.7694	4.4389	5307.2	15.9383
EfficientNet-CICA	4.0358	0.7694	4.4253	5353.2	15.9383

FLOPs are estimated for a 224 × 224 input. Latency was measured on a single NVIDIA GeForce RTX 4090 GPU.

**Figure 4:**
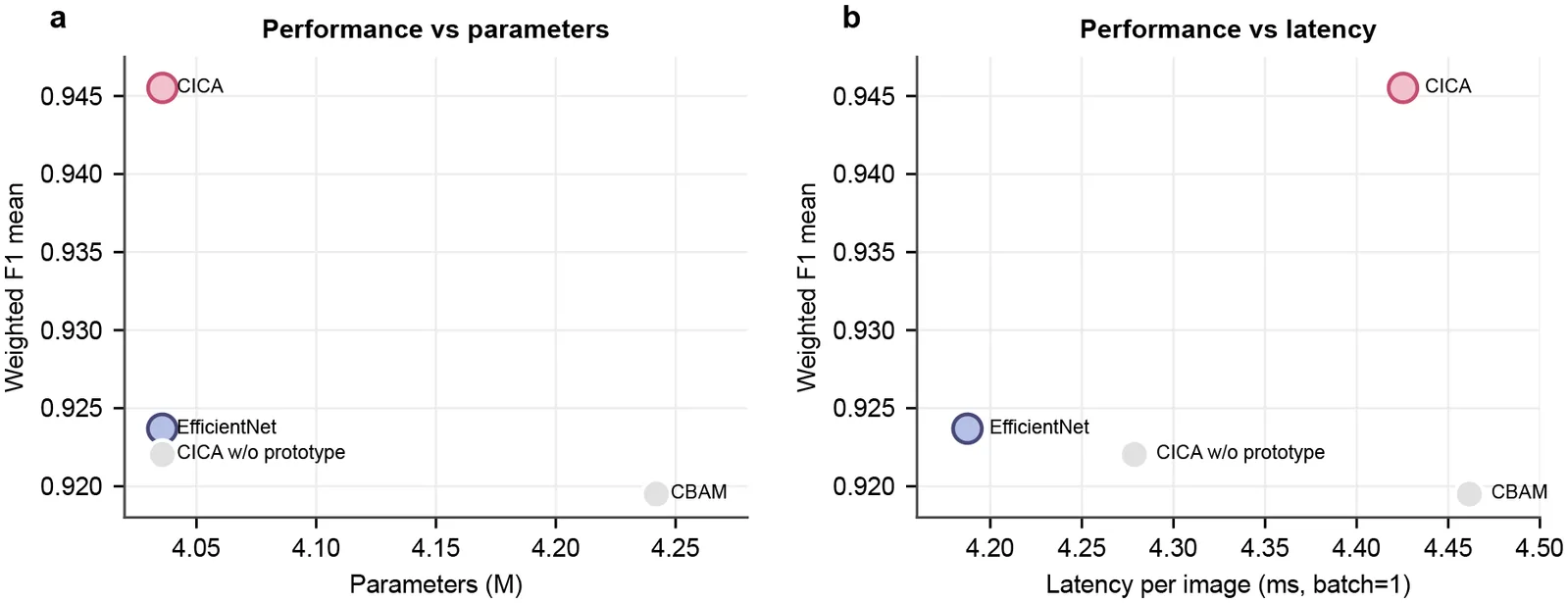
Efficiency-performance trade-off among EfficientNet attention variants. The proposed CICA module improves classification performance with only a small increase in computational cost.

### 3.4 Convergence behavior and similar-class error analysis

To clarify whether 30 training epochs were sufficient for shallow baselines, we plotted validation loss and weighted F1-score curves for VGG, InceptionV3, EfficientNet, and EfficientNet-CICA. As shown in **Figure 5**, VGG and InceptionV3 continued to show slower and more fluctuating convergence than EfficientNet-based models, whereas EfficientNet-CICA reached higher validation F1-score with lower validation loss.

**Figure 5:**
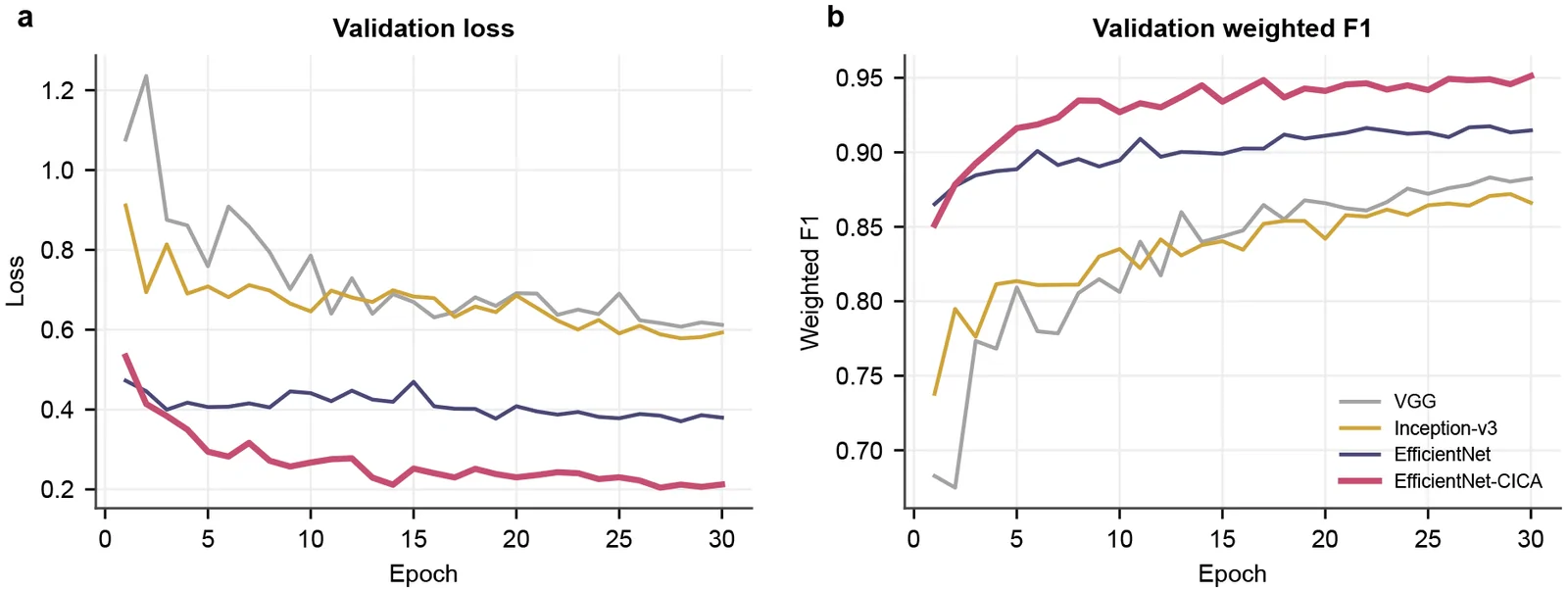
Validation loss and weighted F1-score curves over 30 epochs for VGG, InceptionV3, EfficientNet, and EfficientNet-CICA.

We also reported per-class precision, recall, and F1-score for visually similar sports categories, including tennis/table tennis, hockey/ice hockey, and wrestling/WWE. As summarized in **Table 10** and **Figure 6**, EfficientNet-CICA improved the mean F1-score of hockey from 0.8815 to 0.9173, table tennis from 0.9043 to 0.9350, tennis from 0.9104 to 0.9309, wrestling from 0.9164 to 0.9475, and WWE from 0.8969 to 0.9438 when compared with the EfficientNet baseline. 

**Table 10:** Per-class F1-score for visually similar sports categories.

Category	EfficientNet-CICA	EfficientNet	MedMamba
Hockey	0.9173 ± 0.0307	0.8815 ± 0.0231	0.9084 ± 0.0312
Ice hockey	0.9653 ± 0.0067	0.9511 ± 0.0171	0.9696 ± 0.0123
Table tennis	0.9350 ± 0.0154	0.9043 ± 0.0141	0.9447 ± 0.0126
Tennis	0.9309 ± 0.0150	0.9104 ± 0.0187	0.9368 ± 0.0041
Wrestling	0.9475 ± 0.0214	0.9164 ± 0.0222	0.9273 ± 0.0326
WWE	0.9438 ± 0.0133	0.8969 ± 0.0228	0.9169 ± 0.0312

Values are reported as mean ± standard deviation across five seeds.

**Figure 6:**
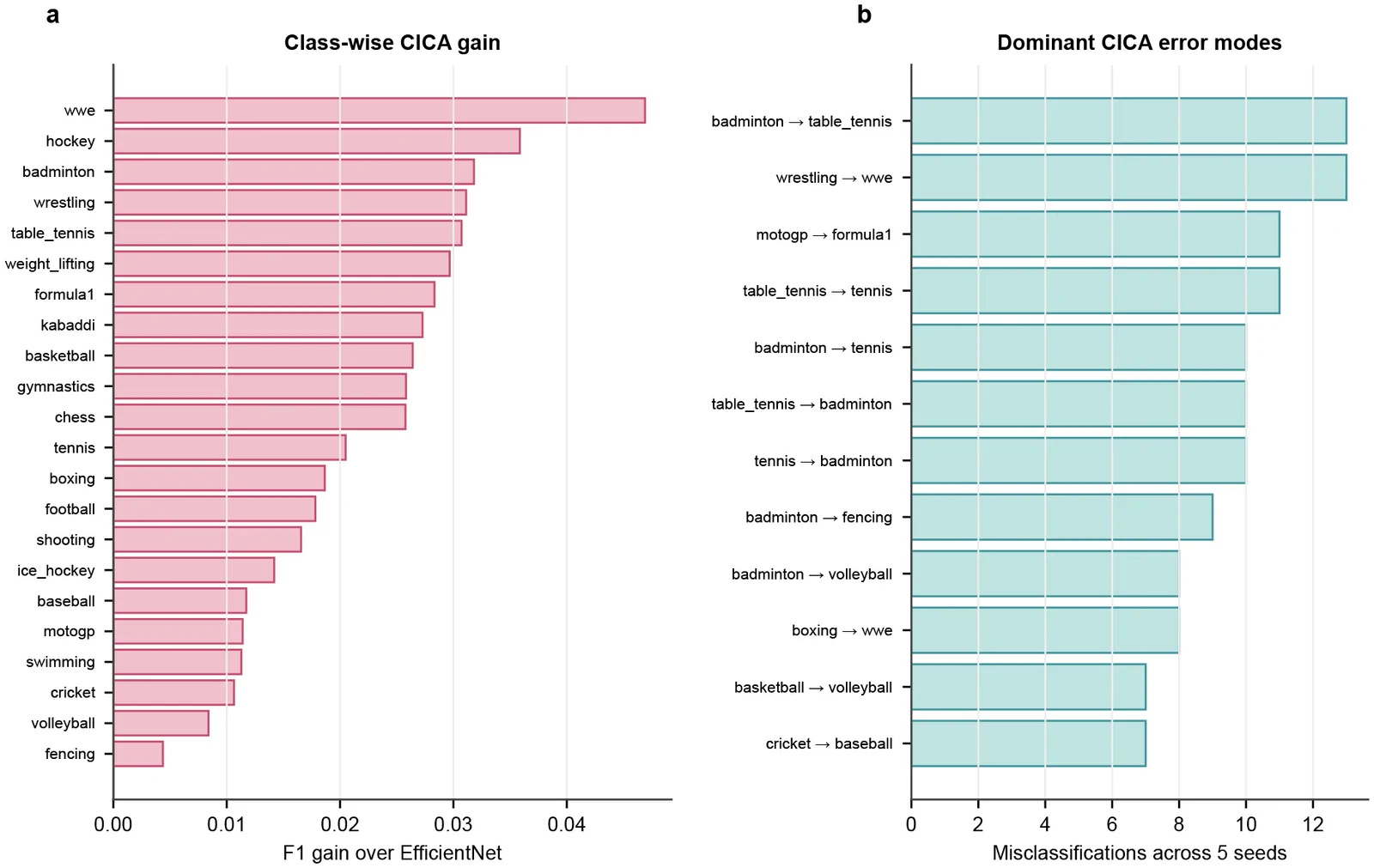
Per-class error analysis for visually similar sports categories. The figure summarizes class-wise performance and common confusion patterns used to interpret residual errors.

### 3.5 Auxiliary physiological relevance analysis

Because the main study is a static-image classification study, we added two auxiliary analyses to clarify the physiological relevance without overstating the scope of the model. First, we designed a toy sport-aware rule-routing demonstration. The 22 sport classes were grouped into illustrative physiologicalrouting categories, including static/low-intensity, technical intermittent, dynamic moderate-to-high intensity, combat/strength-related, and motor-sport activities. For each test image, the predicted sport class was mapped to its routing group and compared with the routing group derived from the ground-truth label. 

As shown in **Table 11**, EfficientNet-CICA improved routing agreement from 0.9521 ± 0.0018 to 0.9628± 0.0042 and reduced under-routing from 0.0217 ± 0.0023 to 0.0176 ± 0.0023. This result does not validate a physiological outcome, but it shows that more accurate sport recognition can reduce errors in a downstream sportaware threshold-selection or model-routing pipeline.

**Table 11:** Toy demonstration of sport-aware physiological rule routing.

Model	Sport accuracy	Routing agreement	Under-routing
EfficientNet	0.9237 ± 0.0024	0.9521 ± 0.0018	0.0217 ± 0.0023
EfficientNet-CICA	0.9456 ± 0.0060	0.9628 ± 0.0042	0.0176 ± 0.0023

Values are reported as mean ± standard deviation across five seeds.

Second, we performed a supplementary PAMAP2 (Reiss and Stricker, 2012) heart-rate regression experiment using wearable IMU features. We compared heart-rate prediction using IMU features alone with prediction using IMU features plus activity type. The results of the supplementary PAMAP2 heart-rate regression experiment are summarized in **Table 12**. Adding activity type reduced MAE from 4.39 to 3.91 bpm and RMSE from 8.14 to 7.02 bpm, while increasing *R*^2^ from 0.921 to 0.941 and Pearson correlation from 0.960 to 0.971. The percentage of predictions within 10 bpm increased from 88.93% to 90.19%. These results provide auxiliary evidence that activity context can improve physiological signal modeling. However, the PAMAP2 experiment should be interpreted as supporting the general importance of activity context, not as direct evidence that static sports images alone can predict physiological signals. 

**Table 12:** Auxiliary PAMAP2 heart-rate regression experiment. The task predicts heart rate from wearable IMU features, with or without activity type as an additional contextual variable.

Setting	MAE (bpm)	RMSE (bpm)	*R* ^2^	Pearson *r*	MAPE (%)	Within 5 bpm (%)
IMU only	4.39	8.14	0.921	0.960	3.85	76.04
IMU + activity type	3.91	7.02	0.941	0.971	3.40	78.15

### 3.6 Visualization comparison

To further evaluate the discriminative ability of the proposed CICA module, we compare EfficientNet and EfficientNet-CICA using confusion matrices, ROC curves, and precision-recall curves. These visualization results provide a more intuitive understanding of how the proposed module improves multi-class sports image classification. **Figure 7** shows the row-normalized confusion matrices of EfficientNet and EfficientNet-CICA. Compared with the original EfficientNet, EfficientNet-CICA obtains stronger diagonal responses for most sports categories, indicating that more samples are correctly classified into their corresponding classes. Meanwhile, the off-diagonal confusion responses are reduced, suggesting that the proposed CICA module can alleviate misclassification between visually similar sports categories. This improvement demonstrates that CICA enhances the category-discriminative representation ability of EfficientNet.

**Figure 7:**
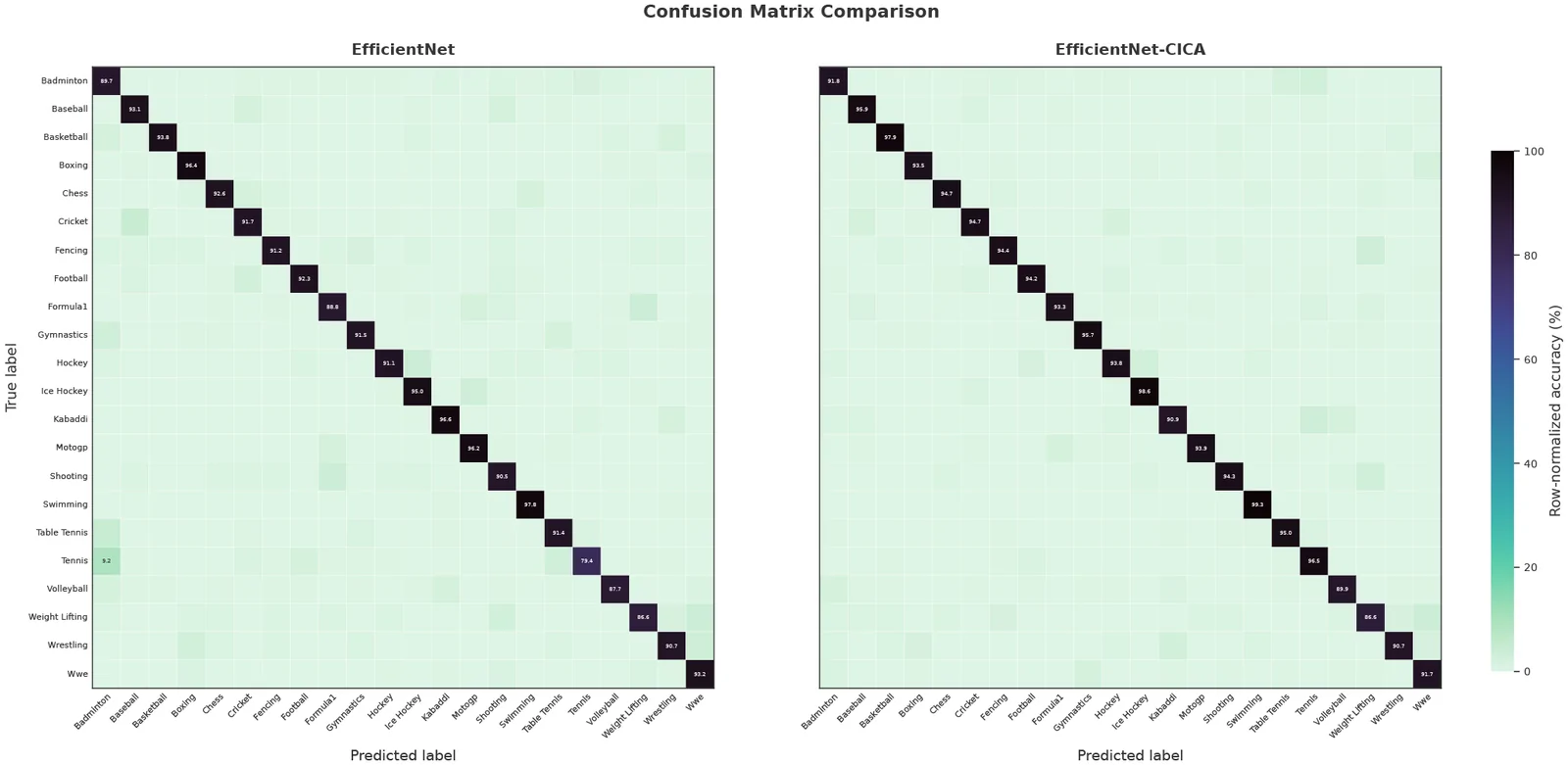
Confusion matrix comparison between EfficientNet and EfficientNet-CICA on the 22-class test set. Rows represent true labels and columns represent predicted labels. Values are row-normalized percentages, so stronger diagonal responses indicate higher class-wise recognition accuracy, whereas offdiagonal responses indicate misclassification between sports categories.

**Figure 8** compares the all-class ROC and precision–recall (PR) curves of the original EfficientNet and EfficientNet-CICA using the original reported prediction outputs. The left column presents ROC curves, whereas the right column presents PR curves. The first row corresponds to the original EfficientNet, and the second row corresponds to EfficientNet-CICA. Each panel includes the curves of all 22 sports categories, while the black dashed curve and boxed annotation indicate the micro-average result. EfficientNet-CICA shows consistently stronger overall discrimination than the baseline. Specifically, the micro-average AUC increases from 0.9966 for the original EfficientNet to 0.9982 after introducing CICA. Similarly, the micro-average AP improves from 0.9685 to 0.9820. The ROC curves of EfficientNet-CICA are generally closer to the upper-left corner, and its PR curves maintain higher precision over a broad recall range. These results indicate that the CICA module improves both class separability and the reliability of prediction confidence across the 22 sports categories.

**Figure 8:**
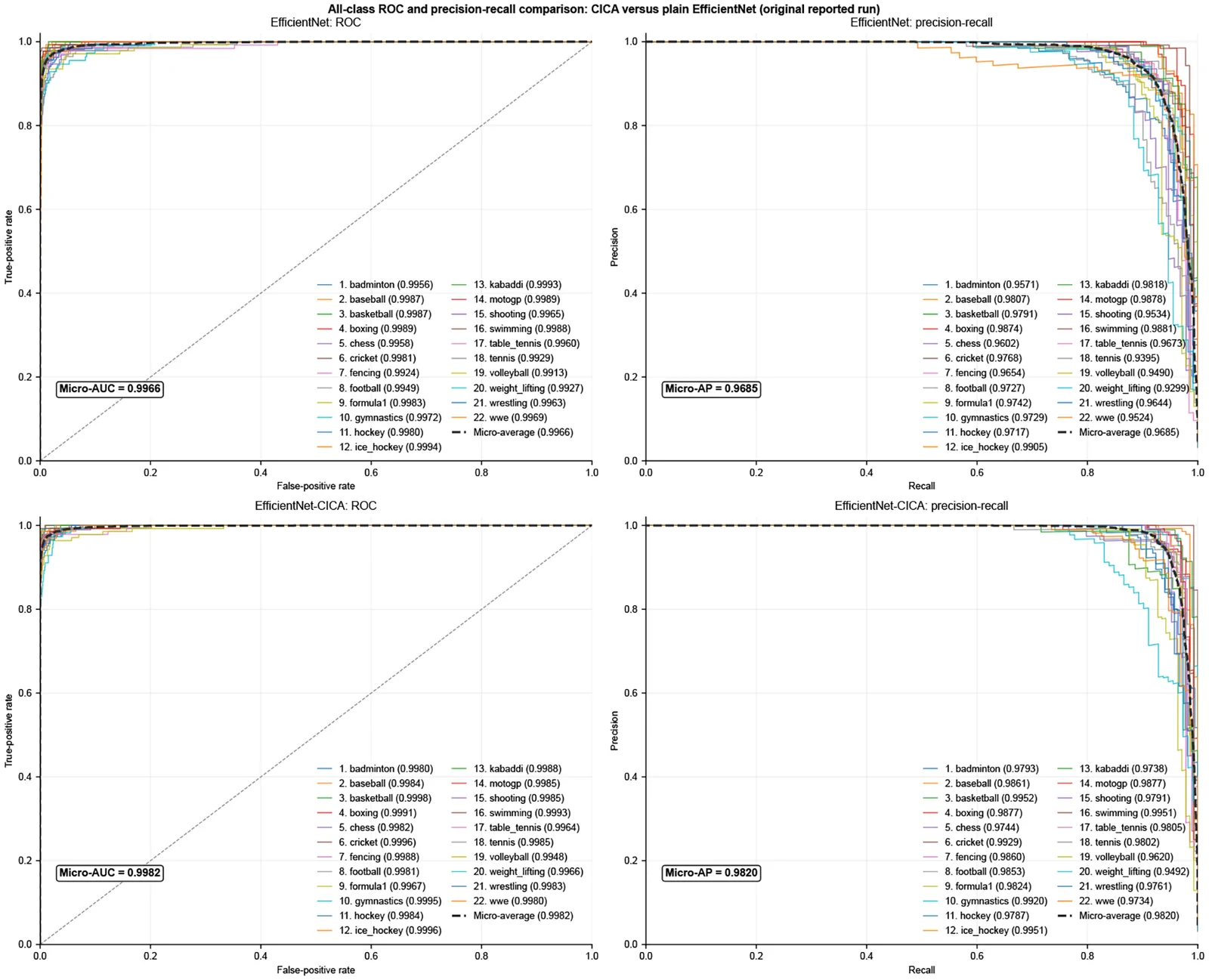
All-class receiver operating characteristic (ROC) and precision-recall (PR) comparison between EfficientNet and EfficientNet-CICA. The left column shows ROC curves and the right column shows PR curves; the first row reports EfficientNet and the second row reports EfficientNet-CICA. Class-wise curves are shown together with micro-average AUC or average precision values, enabling visual comparison of separability and confidence across the 22 sports categories.

To analyze the feature representation ability of the proposed method, **Figure 9** compares the t-SNE distributions of the original EfficientNet baseline and EfficientNet-CICA (Allaoui et al., 2025). Compared with the baseline model, the feature distribution produced by EfficientNet-CICA shows more compact intra-class clusters and clearer inter-class boundaries. In the baseline feature space, several categories exhibit scattered distributions and partial overlap, indicating that the original EfficientNet may still be affected by background similarity and irrelevant local textures. After introducing the CICA module, samples from the same category are more tightly grouped, while different categories are more clearly separated. This demonstrates that CICA can enhance category-aware feature learning by modeling the interaction between class semantic information and channel-wise representations. As a result, the network is encouraged to focus more on sports-related discriminative regions, such as athletes, equipment, and characteristic action patterns, rather than background noise.

**Figure 9:**
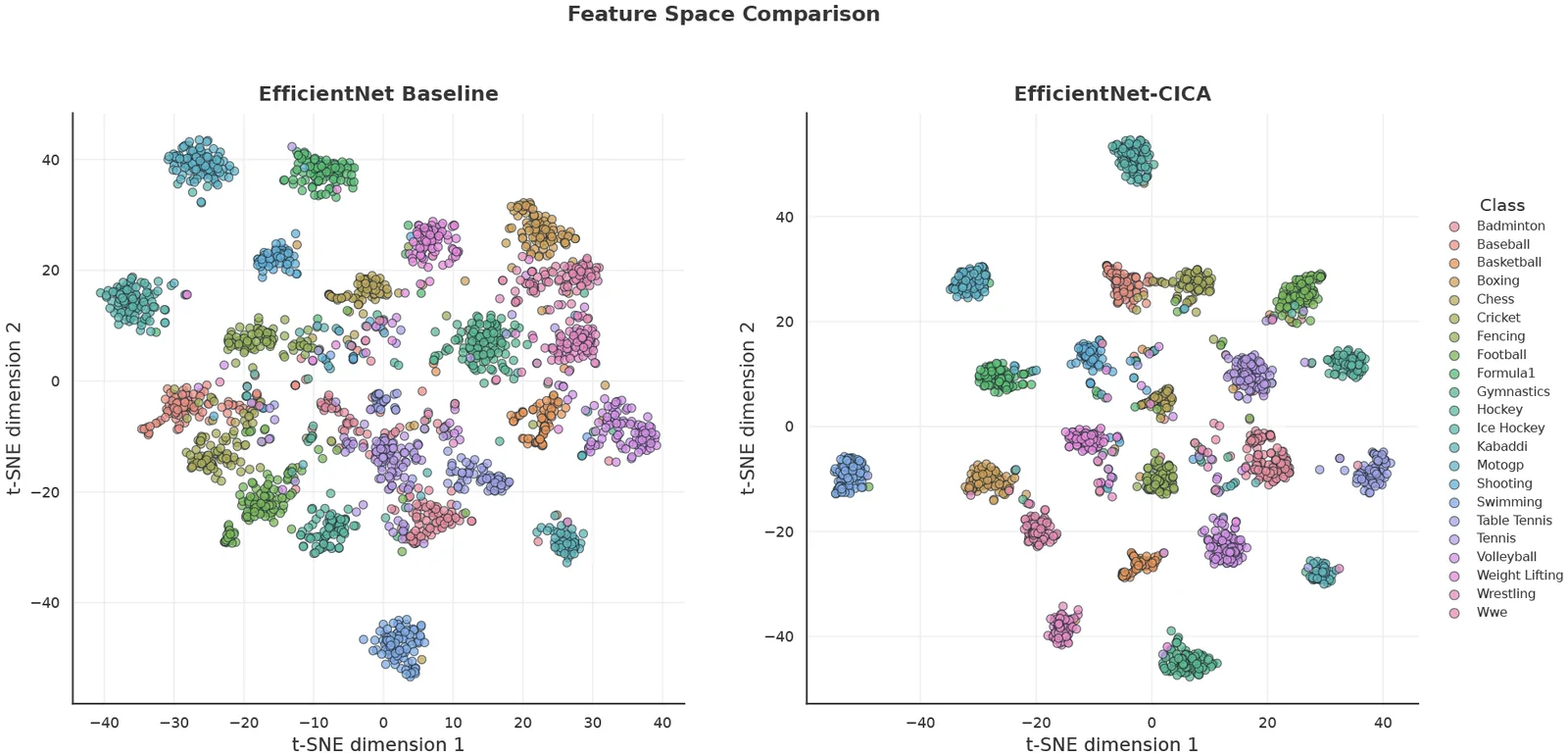
t-SNE feature space comparison between the EfficientNet baseline and EfficientNet-CICA. Each point represents an image sample in the learned feature space, and colors denote sports categories. More compact within-class clusters and clearer between-class separation indicate stronger discriminative feature learning.

### 3.7 Grad-CAM visualization analysis

To further analyze the interpretability of the proposed CICA module, Grad-CAM visualization is performed on both the original EfficientNet and the EfficientNet-CICA model (Selvaraju et al., 2017). As shown in **Figure 10**, both models can identify sports-related regions to some extent. However, the attention responses of the original EfficientNet are relatively dispersed and often highlight broader regions that include background or less discriminative patterns. The heatmaps in **Figure 10a** show that activations spread across multiple areas in the image, with less intensity on key objects such as athletes or sports equipment. In contrast, after integrating the proposed CICA module, EfficientNet-CICA produces more concentrated and semantically meaningful activation regions, as shown in **Figure 10b**. The heatmaps indicate stronger activation on the main athletes, balls, or equipment, while suppressing irrelevant background regions. For instance, in images of basketball and football, the model emphasizes the players’ bodies and motion areas more precisely. Similarly, for tennis and table tennis, the rackets and balls are clearly highlighted. The intensity distribution in the heatmaps also demonstrates that the proposed module encourages the network to focus on category-discriminative features with higher confidence. Overall, the Grad-CAM results confirm that integrating the CICA module not only improves classification performance but also enhances the interpretability of the model. By producing sharper, more focused attention maps that align with human-recognizable sports cues, the network becomes more transparent in its decision-making process, and the highlighted regions in the heatmaps can serve as reliable indicators of the visual evidence leveraged for recognition. To avoid showing only successful examples, we additionally inspected representative failure cases. As shown in **Figure 11**, residual errors often occurred when two categories shared equipment, court structure, body-contact patterns, or visually similar backgrounds. These failure cases indicate that CICA improves attention concentration but does not eliminate ambiguity in static images, especially when temporal motion cues are absent.

**Figure 10:**
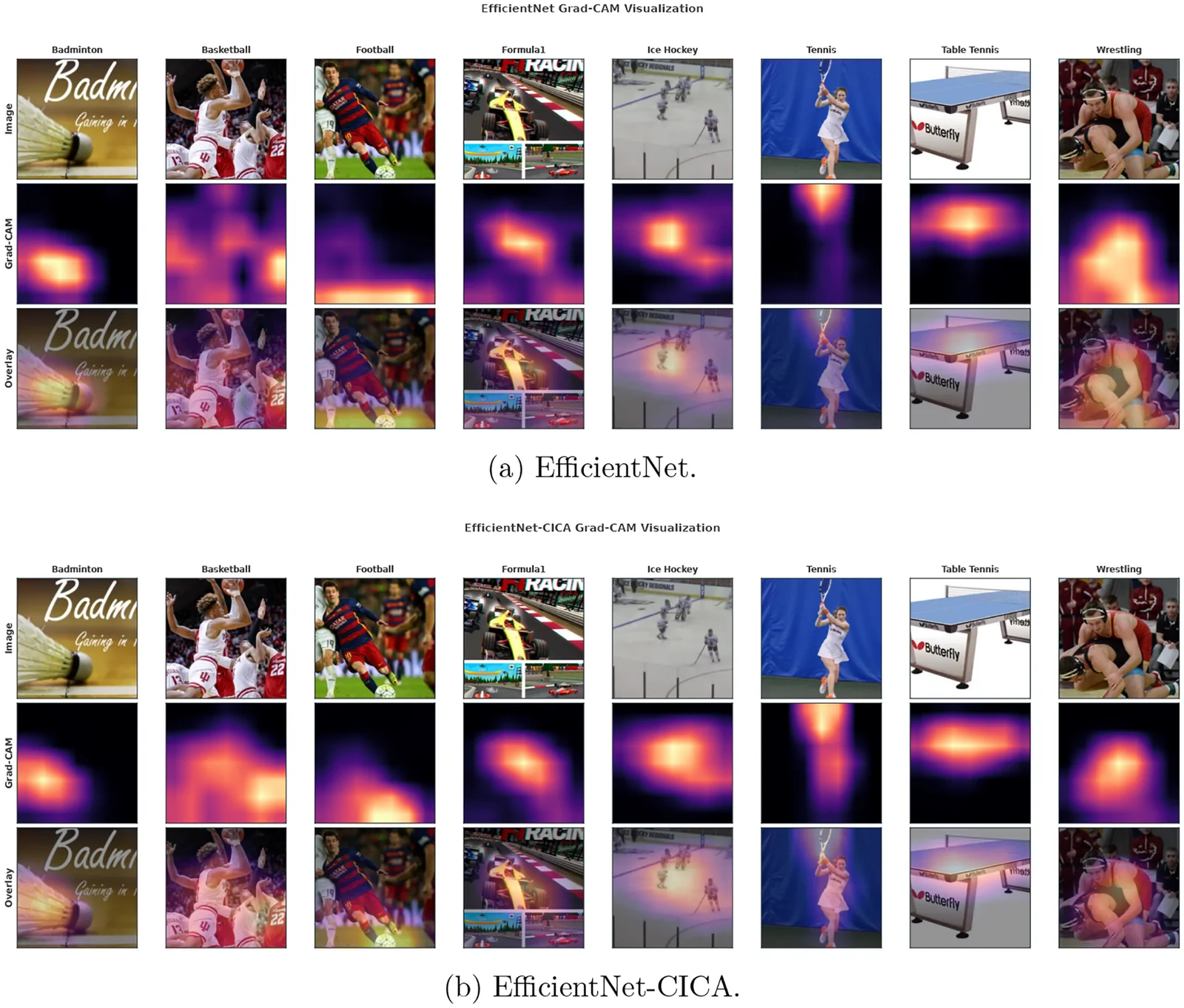
Grad-CAM visualization comparison between EfficientNet and EfficientNet-CICA. Panels **(a, b)** correspond to EfficientNet and EfficientNet-CICA, respectively. Within each panel, columns show representative sports categories, and rows show the original image, Grad-CAM heatmap, and heatmap overlay. Compared with the baseline, EfficientNet-CICA produces more focused and semantically meaningful activation regions over athletes, equipment, balls, or sport-specific action areas, indicating improved visual interpretability in addition to classification performance.

**Figure 11:**
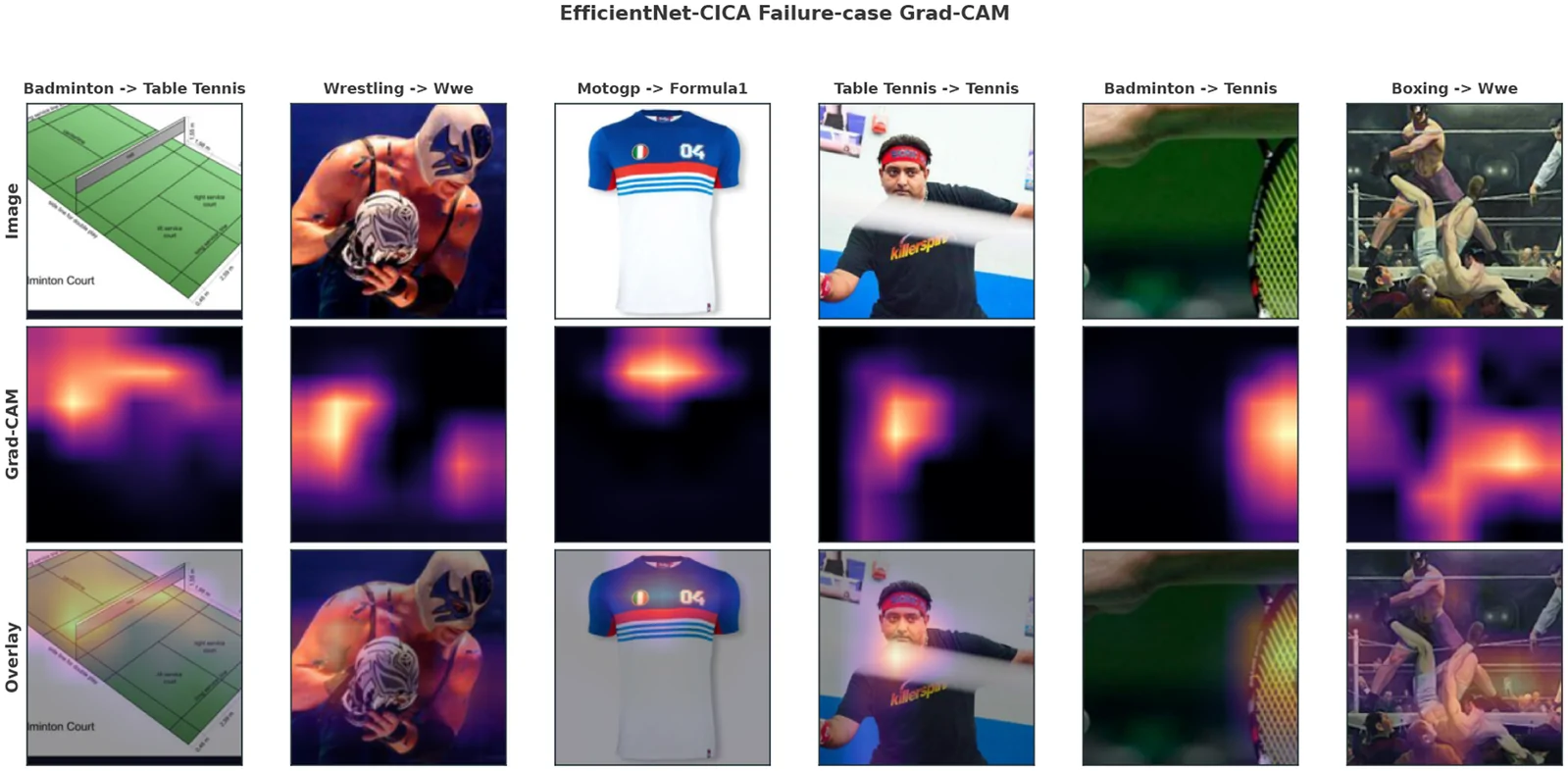
Representative Grad-CAM failure cases for EfficientNet-CICA. The examples show residual confusions between visually similar categories, illustrating that static-image recognition remains limited when categories share similar equipment, field/court layouts, or body-contact patterns.

### 3.8 Prototype selection strategy analysis

To further investigate whether the improvement of CICA comes from meaningful prototype-guided counterfactual intervention, we compare different prototype selection strategies, including no prototype, random negative prototype, ground-truth positive prototype, and most similar negative prototype. The results are reported in **Table 6**. As shown in **Table 6**, the no-prototype variant achieves an accuracy of 0.9211, a weighted F1-score of 0.9211, and a macro AUC of 0.9969. When a random negative prototype is used, the performance remains almost unchanged, with an accuracy of 0.9211, a weighted F1-score of 0.9210, and a macro AUC of 0.9970. This indicates that simply introducing a negative category prototype does not necessarily provide effective semantic guidance. Since the randomly selected prototype may be semantically distant from the current sample, it is unable to construct a sufficiently informative counterfactual contrast for distinguishing visually similar sports categories.

When the ground-truth positive prototype is used, the accuracy increases to 0.9376 and the weighted F1-score reaches 0.9375, showing that category-level prototype information can provide useful semantic guidance for channel recalibration. However, this strategy is still inferior to the most similar negative prototype strategy. This is because the positive prototype mainly reinforces the current class representation, but does not explicitly model the difference between the current sample and its visually confusing categories.

The proposed most similar negative prototype strategy achieves the best performance, with an accuracy of 0.9405, a weighted F1-score of 0.9404, and a macro AUC of 0.9982. Compared with the no-prototype variant, it improves the accuracy by 1.94 percentage points and the weighted F1-score by 1.93 percentage points. Compared with the ground-truth positive prototype strategy, it further improves the accuracy by 0.29 percentage points. These results demonstrate that the key advantage of CICA lies in hard negative semantic intervention. By selecting the most similar negative prototype, CICA constructs a more discriminative counterfactual descriptor, forcing the model to focus on channels that distinguish the current sports category from visually similar categories.

Overall, the prototype selection strategy analysis verifies that the proposed CICA module benefits not only from category prototype guidance, but more importantly from the use of confusing negative prototypes for counterfactual channel intervention. This supports the motivation that channel attention should be guided by category-discriminative semantic contrast rather than relying solely on activation statistics.

## 4 Discussion

### 4.1 Physiological motivation and scope

The present study should primarily be interpreted as a static-image sports-context classification study. The main experiment does not directly measure heart rate, oxygen uptake, fatigue, biomechanical loading, or wearable signals. Therefore, our physiological claim is intentionally limited: sport type may serve as an activity-context variable for downstream physiological interpretation, but direct physiological validity requires additional multimodal experiments. The two auxiliary analyses partly address this motivation. The toy rule-routing demonstration showed that improved sport recognition reduced errors in selecting an illustrative sport-aware interpretation rule. The PAMAP2 experiment further showed that adding activity type to IMU features improved heart-rate regression, reducing MAE from 4.39 to 3.91 bpm. Together, these results suggest that activity context can influence physiological modeling, but they do not prove that static images alone are sufficient for physiological monitoring.

### 4.2 Significance of the proposed model

The proposed EfficientNet-CICA model provides an accurate and interpretable visual module for sportscontext recognition. In context-aware exercise monitoring, incorrect recognition of the sport category may lead to inappropriate interpretation of physiological or wearable signals. Therefore, the recognition model should not only achieve high classification performance, but also reduce confusion between visually similar sports categories. The CICA module addresses this requirement by introducing class prototypes and counterfactual channel intervention. By comparing the current feature descriptor with a visually similar negative prototype, the model is encouraged to focus on category-discriminative cues that distinguish the current sport from confusing alternatives. The ablation results further show that meaningful prototype-guided intervention is more effective than standard attention mechanisms or random prototype selection. In addition, the Grad-CAM results showed that EfficientNet-CICA focused more consistently on athletes, equipment, body-action regions, and sport-specific environments. These cues are closely related to movement patterns, athlete–equipment interaction, and activity-specific demands. Thus, the proposed model may serve as a visual-context annotation component in future systems that integrate images with physiological, biomechanical, or wearable measurements. This statement is a future application hypothesis rather than a direct conclusion from physiological outcome testing.

### 4.3 Limitations and future directions

Several limitations should be considered. First, the study used a single public sports image dataset. Although the dataset contains 22 categories and 14,192 valid images, additional validation on larger and more diverse datasets is needed to assess cross-domain generalization. Second, the current model uses static images and does not capture temporal movement dynamics, which are highly relevant to exercise physiology. Extending the framework to video-based recognition, action localization, and temporal movement analysis would make it more suitable for exercise and sport monitoring. Third, CICA relies on prototype estimation during training. When the number of samples is limited or class imbalance is severe, prototypes may become unstable. More robust prototype learning strategies, including adaptive updating, multiple prototypes per class, and contrastive prototype optimization, should be explored. Fourth, the auxiliary PAMAP2 analysis supports the usefulness of activity context for heart-rate prediction, but it does not directly connect the image classifier to wearable heart-rate prediction on synchronized visual–physiological recordings. Future work should therefore collect or use datasets containing synchronized sport images/videos, wearable signals, and physiological outcomes. Finally, further improvements from the perspective of deep learning architectures should be explored. Advanced strategies may further enhance the recognition performance and generalization capability of the proposed framework (Yan et al., 2025; Zhang et al., 2026). These improvements could facilitate more accurate and robust visual-context understanding for intelligent sports analysis and physiological monitoring applications.

## 5 Conclusion

This study proposed an EfficientNet-CICA framework for category-aware visual recognition of sports contexts. By introducing class prototype information and counterfactual channel intervention, the proposed module enhanced discriminative channel responses and improved the model’s ability to focus on athletes, sports equipment, actions, and sport-specific visual structures. Experiments on a 22-class public sports image dataset showed that EfficientNet-CICA achieved higher accuracy and weighted F1-score than representative CNN, Transformer, and state space model baselines. Ablation experiments and visualization analyses further confirmed the effectiveness of prototype-guided intervention, demonstrating that the proposed method improved feature separability and paid greater attention to sport-relevant key regions. Accurate sports-context recognition may provide useful activity-context information for future multimodal exercise physiology systems, but the present work does not directly validate physiological outcomes from images. The added toy routing and PAMAP2 analyses suggest that activity context can affect downstream physiological rule selection and heart-rate modeling. Future work should test the proposed visual-context recognition module using synchronized physiological, biomechanical, wearable, and visual data.

## Data Availability

The original contributions presented in the study are included in the article/supplementary material. Further inquiries can be directed to the corresponding author.
